# Licochalcone A inhibits EGFR signalling and translationally suppresses survivin expression in human cancer cells

**DOI:** 10.1111/jcmm.16135

**Published:** 2020-11-27

**Authors:** Feng Gao, Ming Li, Xinfang Yu, Wenbin Liu, Li Zhou, Wei Li

**Affiliations:** ^1^ Department of Ultrasonography The Third Xiangya Hospital of Central South University Changsha China; ^2^ Cell Transplantation and Gene Therapy Institute The 3rd Xiangya Hospital of Central South University Changsha China; ^3^ Changsha Stomatological Hospital Changsha China; ^4^ Department of Cancer Biology Lerner Research Institute Cleveland Clinic Cleveland OH USA; ^5^ Department of Pathology Hunan Cancer Hospital Changsha China; ^6^ Department of Pathology Xiangya Hospital of Central South University Changsha China; ^7^ Department of Radiology The Third Xiangya Hospital of Central South University Changsha China

**Keywords:** epidermal growth factor receptor, licochalcone A, non‐small‐cell lung cancer, survivin

## Abstract

Dysfunction of epidermal growth factor receptor (EGFR) signalling plays a critical role in the oncogenesis of non–small‐cell lung cancer (NSCLC). Here, we reported the natural product, licochalcone A, exhibited a profound anti‐tumour efficacy through directly targeting EGFR signalling. Licochalcone A inhibited in vitro cell growth, colony formation and in vivo tumour growth of either wild‐type (WT) or activating mutation EGFR‐expressed NSCLC cells. Licochalcone A bound with L858R single‐site mutation, exon 19 deletion, L858R/T790M mutation and WT EGFR ex vivo, and impaired EGFR kinase activity both in vitro and in NSCLC cells. The in silico docking study further indicated that licochalcone A interacted with both WT and mutant EGFRs. Moreover, licochalcone A induced apoptosis and decreased survivin protein robustly in NSCLC cells. Mechanistically, we found that treatment with licochalcone A translationally suppressed survivin through inhibiting EGFR downstream kinases ERK1/2 and Akt. Depletion of the translation initiation complex by eIF4E knockdown effectively inhibited survivin expression. In contrast, knockdown of 4E‐BP1 showed the opposite effect and dramatically enhanced survivin protein level. Overall, our data indicate that targeting survivin might be an alternative strategy to sensitize EGFR‐targeted therapy.

## 1 INTRODUCTION

Non–small‐cell lung cancer (NSCLC) is the most common cause of cancer death worldwide. The identification of driver mutations, such as somatic mutations in the epidermal growth factor receptor (EGFR) gene, and the subsequent development of EGFR tyrosine kinase inhibitors (TKIs) as targeted therapy have significantly revolutionized the treatment landscape of these tumours.^1^, ^2^, ^3^ Osimertinib (AZD9291) is an orally administered third‐generation EGFR‐TKI that selectively targets EGFR activation mutations, including L858R point mutations, the deletions in exon 19 and T790M mutation.^4^, ^5^, ^6^, ^7^ Although osimertinib has shown a robust clinical response, most patients eventually develop acquired resistance to this treatment.^5^, ^7^ Thus, further elucidate the molecular mechanisms of osimertinib resistance or develop novel approaches to counteract resistance is an urgent demand in clinic treatment.

Many chemotherapy drugs exhibited adverse side effects. Identification of new anti‐tumour chemicals from natural compounds might be a safer alternative for anti‐tumour treatment. Licochalcone A (Lico A), a flavonoid extracted from licorice root, exerts a wide range of pharmacological effects in the treatment of human diseases, including inflammation, infections and gastric ulcers. Recently, the in vitro and in vivo studies have demonstrated that licochalcone A inhibits multiple human solid tumours,^8^ including lung,^9^ gastric,^10^ prostate,^11^ liver^8^ and ovarian cancer.^12^ Licochalcone A promotes cell‐cycle arrest, induces apoptosis, reduces angiogenesis and metastasis, etc.^13^, ^14^, ^15^ However, the direct targets of licochalcone A in human cancer cells have not been elaborated, and the effect of licochalcone A on EGFR signalling has not been reported.

In the present study, we demonstrated that licochalcone A suppressed the activation of both wild‐type and mutant EGFRs, and translationally suppressed survivin expression in NSCLC cells. The specific targeting of the EGFR‐survivin axis might provide opportunities for NSCLC prevention and treatment.

## 2 MATERIALS AND METHODS

### 2.1 Reagents and cell culture

The chemicals for molecular biology and buffer preparation, such as Tris, NaCl, SDS and licochalcone A (>98%), were obtained from Sigma. Osimertinib, PD98059 and MK2206 were purchased from Selleck Chemicals. Antibodies against ERK1/2 (#9102), p‐ERK1/2‐Thr202/Tyr204 (#4370EGFR (#4267), PARP (#9532), Akt (#4691), β‐actin (#3700), survivin (#2808), 4E‐BP1 (#9644)), p‐EGFR‐Tyr1068 (#3777), p‐Akt‐Ser473 (#4060), Bcl‐2 (#15071), cleaved caspase 3 (#9664), Bcl‐xL (#2764) and Mcl‐1 (#94296) were purchased from Cell Signaling Technology, Inc. Anti‐eIF4E (LS‑B12932) antibody was obtained from LifeSpan BioSciences, Inc. Human NSCLC cells, including H3255 (EGFR L858R), HCC827 (EGFR Del E746‐A750), H1975 (EGFR L858R/T790M) and A549 (EGFR WT), and immortalized lung epithelial or fibroblast cells, such as HBE, MRC5 and NL20, were obtained from American Type Culture Collection (ATCC, Manassas, VA). Cell culture was performed following the standard protocols provided by ATCC. All cells were authenticated and cytogenetically tested before being frozen. The foetal bovine serum (FBS) and cell culture medium were products of Thermo Fisher Scientific. The Ba/F3 cell was purchased form Cell Engineering Division/RIKEN BioResource Center and maintained according to the instructions provided.

### 2.2 Western blot analysis

For immunoblotting (IB) analysis, whole‐cell extracts were prepared using RIPA buffer (10 mmol/L Tris‐Cl (pH 8.0), 0.5 mmol/L EGTA, 0.1% SDS, 1 mmol/L EDTA, 0.1% sodium deoxycholate, 1% Triton X‐100 and 140 mmol/L NaCl).^16^ The BCA protein assay (Thermo Fisher Scientific) was used for protein concentration. Protein sample was boiled with SDS‐PAGE loading buffer at and subjected to SDS‐PAGE gel electrophoresis.

### 2.3 MTS assay

Cells were suspended at the concentration of 2 × 10^4^, and 100 μL of aliquot was seed into 96‐well plates, followed by incubation with licochalcone A or osimertinib at various time‐points. Cell viability was analysed with MTS assay (Promega).

### 2.4 Lentiviral package and stable lines generation

The EGFR cDNA clones, including L858R EGFR, L858R/T790M EGFR, Del E746‐A750 EGFR and wild‐type EGFR, were obtained from Origene and used for virus package. For virus infection, polybrene (8 μg/mL) and virus supernatant were added to cell culture medium and maintained for 24 hours. The infected cells were selected with puromycin (1 μg/mL) for 7 days. The CRISPR‐Cas9–based EGFR knockout was performed with the EGFR sgRNA (TGAGCTTGTTACTCGTGCCT) following the standard protocols.

### 2.5 Anchorage‐independent growth

The colony formation in soft agar was performed as described previously.^17^ Cells were suspended (8000 cells/mL) in 1 mL of 0.3% agar with Eagle's basal medium containing 1% antibiotics, 10% FBS, and osimertinib or different concentrations of licochalcone A overlaid into six‐well plates containing a 0.6% agar base. The cultures were maintained for 2 weeks in a 37°C, 5% CO_2_ incubator.

### 2.6 Flow cytometry

NSCLC cells were treated with osimertinib or licochalcone A and dissociated with trypsin. Flow cytometry analysis was conducted as described previously.^18^ Briefly, cells were washed with PBS and centrifuged, followed by suspending at a final concentration of 1 × 10^6^ cells/mL. The staining buffer which contains propidium iodide and Annexin V (5 µL) was added to the cell suspension and incubated for 15 minutes in the dark. The apoptotic cells were determined with a FACSort Flow Cytometer (BD, San Jose, CA, USA).

### 2.7 In vitro EGFR kinase assay

The recombinant active Del E746‐A750 EGFR, L858R/T790M EGFR, L858R EGFR and WT EGFR were purchased from Millipore. Briefly, the active EGFRs (100 ng) were incubated with 500 μmol/L angiotensin II, osimertinib or licochalcone A for 5 minutes at room temperature. The ATP mixture (0.25 μmol/L ATP and 25 mmol/L MgAc containing 10 μCi [γ‐32P] ATP) was added into the reaction and incubated at 30°C for 15 minutes, and transferred onto P81 papers. The papers were washed subsequently with 0.75% phosphoric acid and acetone. The scintillation counter was used for radioactive incorporation analysis.

### 2.8 ATP competition assay and in vitro pull‐down assay

The ATP competition assay and in vitro pull‐down assay were performed as described previously.^19^ Briefly, licochalcone A‐Sepharose 4B beads or Sepharose 4B beads were incubated with the active kinase with different concentrations of ATP or the NSCLC cell lysate (500 μg) in reaction buffer (150 mmol/L NaCl, 50 mmol/L Tris‐HCl (pH 7.5), 0.01% Nonidet P‐40, 1 mmol/L DTT, 5 mmol/L EDTA, 1 × protease inhibitor mixture, 0.02 mmol/L phenylmethysulphonyl fluoride and 2 μg/mL bovine serum albumin) overnight at 4°C. The beads were then washed with wash buffer for 5 times and subjected to Western blotting analysis.

### 2.9 Molecular modelling

Homology modelling: The three‐dimensional structure of exon 19 deletion mutation (residues 696‐984) EGFR was modelled based on the wild‐type (WT) EGFR crystal structure using Modeller,^20^ and the crystal structure of EGFR (PDB: 4jr3) was used as the template for homology modelling.^21^ Molecular docking: After prepared the structures of L858R EGFR (PDB: 2itv), WT EGFR (PDB: 4jr3), EGFR with exon 19 deletion and L858R/T790M EGFR (PDB: 3w2p), including minimizing heavy atoms, filling in missing side chains and adding hydrogens with Protein Preparation Wizard in Schrödinger Suite 2013, the corresponding protein grid files were generated suitable for docking. Then, all the ligands were well pre‐treated in LigPrep, the docking was performed based on the standard precision mode of Glide. Docking poses and binding modes for each receptor‐ligand complex were analysed, and PyMOL was used for final figures generation.

### 2.10 In vivo tumour growth

The xenograft mouse model was performed following the guidelines of the Medical Research Animal Ethics Committee, Central South University, China. NSCLC cell lines, including H3255 (2 × 10^6^), H1975 (1 × 10^6^), HCC827 cells (2 × 10^6^) and A549 (2 × 10^6^), were suspended in 100 μL RPMI‐1640 medium and inoculated s.c. into the right flank of 6‐week‐old female athymic nude mice. Osimertinib (2 mg/kg) treatment was initiated and repeated every 2 days by oral gavage when tumour volume reached 100 mm^3^. Licochalcone A (10 mg/kg)/vehicle control was administrated by intraperitoneal injection. Tumour volume was calculated following the formula of A (longest diameter) × B (shortest diameter)^2^ × 0.5.

### 2.11 Immunohistochemical (IHC) staining

IHC staining for xenograft tumour tissues was performed as described previously.^22^ Briefly, tissue sections were deparaffinized and rehydrated, followed by immersing into boiling sodium citrate buffer (10 mmol/L, pH 6.0) for 10 minutes for antigen retrieval. The tissue section was washed with PBS twice and incubated with 3% H_2_O_2_ in methanol for 10 minutes. Tissues were blocked with 50% goat serum albumin and incubated with the primary antibody overnight in a humidified chamber at 4°C, followed by hybridizing with the secondary antibody at room temperature for 1 hour. The target protein was visualized with DAB substrate.

### 2.12 Statistical analysis

Statistical analysis was performed with SPSS 16.0 (SPSS, Inc). The quantitative data were expressed as means ± SD. Student's *t* test or one‐way ANOVA was used for significant differences determination. A probability value less than 0.05 was used as the criterion for statistical significance.

## 3 RESULTS

### 3.1 Licochalcone A effectively inhibits the growth of both osimertinib‐sensitive and osimertinib‐resistant NSCLC cells

**Figure 1:**
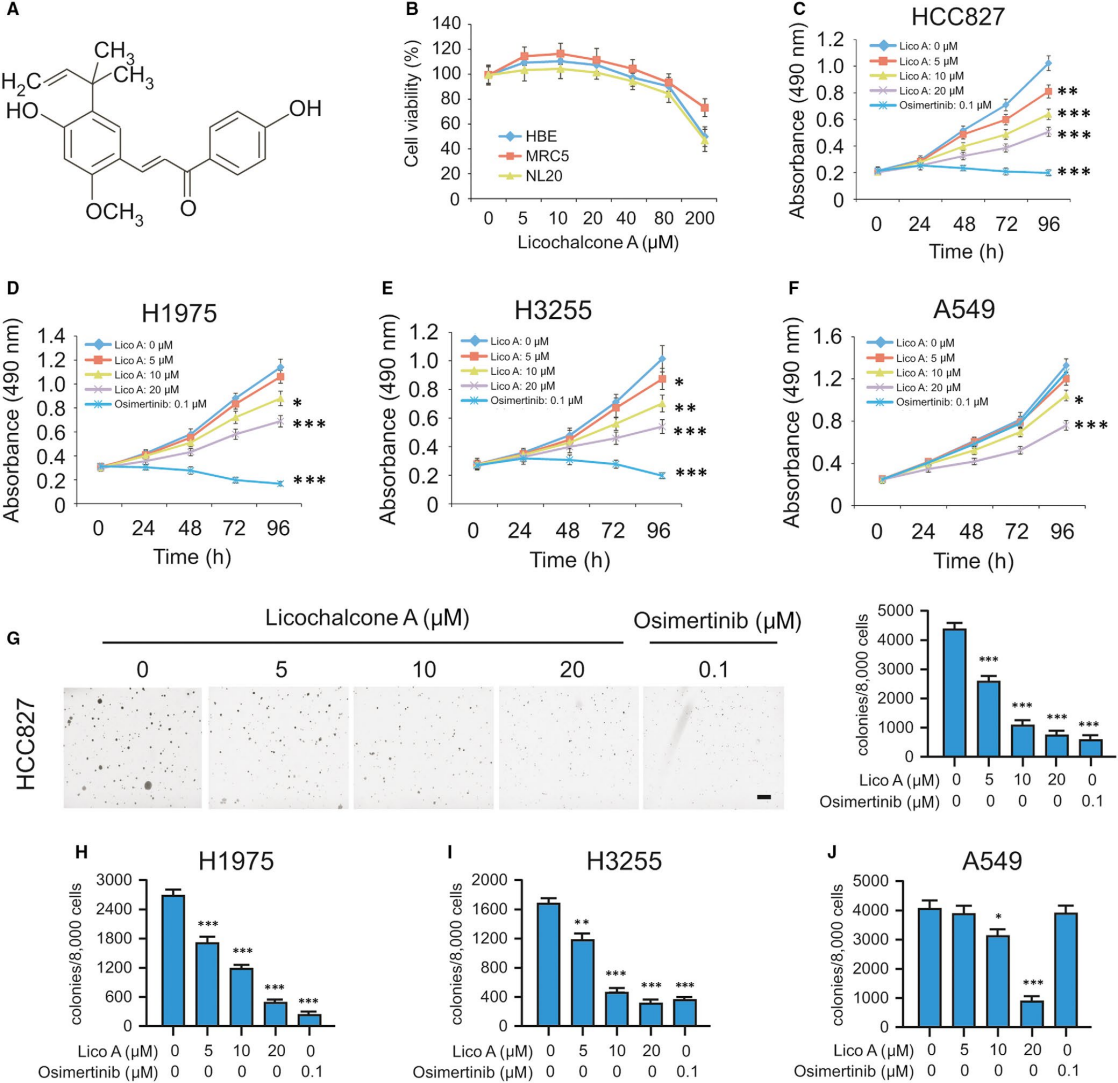
Licochalcone A suppresses non–small‐cell lung cancer (NSCLC) cells. A, The structure of licochalcone A. B, MRC5, NL20 and HBE cells were treated with licochalcone A for 72 h and subjected to MTS assay analysis for cell viability. C‐F, HCC827 (C), H1975 (D), H3255 (E) and A549 (F) cells were treated with licochalcone A or osimertinib and subjected to MTS assay analysis for cell viability. *P < .05, ***P < .01, ***P < .001. Lico A, licochalcone A. G‐J, Colony formation of HCC827 (G), H1975 (H), H3255 (I) and A549 (J) cells with licochalcone A or osimertinib treatment. *P < .05, ***P < .01, ***P < .001. Scale bar, 500 μm

Previous studies have demonstrated that licochalcone A (Figure 1A, MW. 268.26) exerted potent biological functions in multiple human disease models.^23^ However, the inhibitory effect and anti‐tumour mechanism of licochalcone A on NSCLC are still elusive. In the present study, we first investigated whether licochalcone A exerts any cytotoxic effect on immortalized lung epithelial and fibroblast cells, such as HBE, MRC5 and NL20. The result showed that licochalcone A exhibited no significant cytotoxicity against these cells when concentration up to 80 μmol/L (Figure 1B). NSCLC cells which harbour the activating mutations of EGFR, but not the WT EGFR‐expressing A549 cells, were dramatic response to osimertinib treatment. Interestingly, licochalcone A exhibited a significant anti‐tumour efficacy against all of these test NSCLC cells in a time‐ and dose‐dependent manner (Figure 1C‐F). Even 5 µm of licochalcone A had shown little effect on cell growth inhibition, higher concentration (10 or 20 μmol/L) or long‐term (48‐96 hours) exposure to licochalcone A strongly suppressed cell proliferation. Based on these data, we then determined the effect of licochalcone A on colony formation of NSCLC cells. We found that osimertinib significantly decreased the colony number of H3255, HCC827 and H1975 cells as expected, and licochalcone A could also strongly inhibit these three cell lines growth in soft agar even at the concentration of 5 μmol/L. Additionally, licochalcone A, but not osimertinib, markedly suppressed colony formation of A549 cells dose‐dependently (Figure 1G‐J, Figure S1A‐C). These results indicate that licochalcone A suppresses the growth of either WT or mutant EGFR expression NSCLC cells, but has no obvious cytotoxic effect on non‐tumour lung cells.

### 3.2 Licochalcone A binds and inhibits EGFRs activities

**Figure 2:**
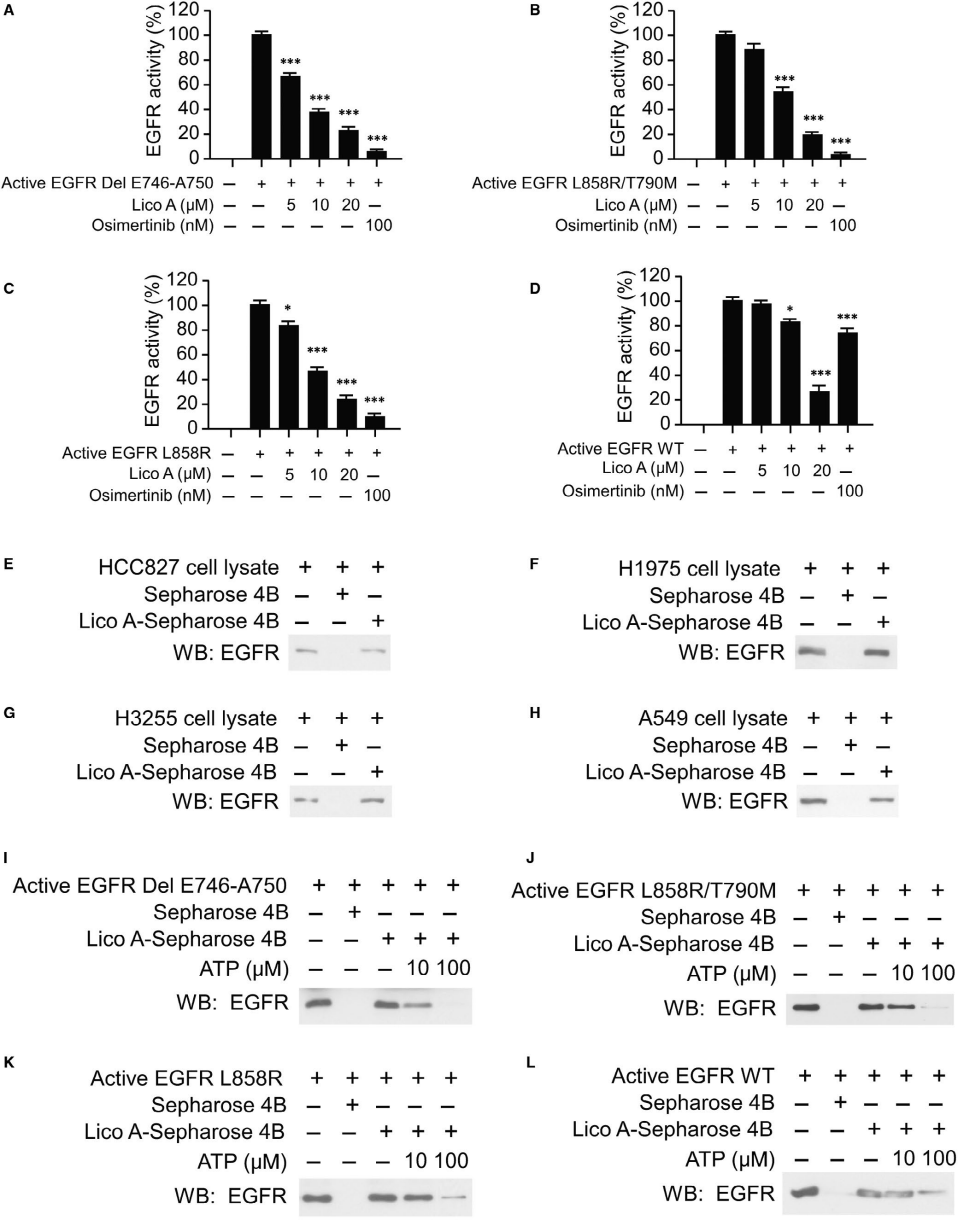
Licochalcone A suppresses the activity of EGFR kinase. A‐D, Licochalcone A inhibits EGFR Del E746‐A750 (A), EGFR L858R/T790M (B), EGFR L858R (C) and EGFR (wild‐type) WT (D) kinase activities. *P < .05, **P < .01, ***P < .001. E‐H, Licochalcone A‐Sepharose 4B beads (Sepharose 4B beads only as control) were incubated with cell lysates (500 μg) from HCC827 (E), H1975 (F), H3255 (G) and A549 (H) overnight at 4°C and subjected to immunoblotting (IB) analysis. I‐L, EGFR Del E746‐A750 (I), EGFR L858R/T790M (J), EGFR L858R (K) and EGFR WT (L) active kinases were incubated with various doses of ATP overnight and bind with licochalcone A‐Sepharose 4B beads for 4 h, and EGFR protein level was determined by IB analysis

**Figure 3:**
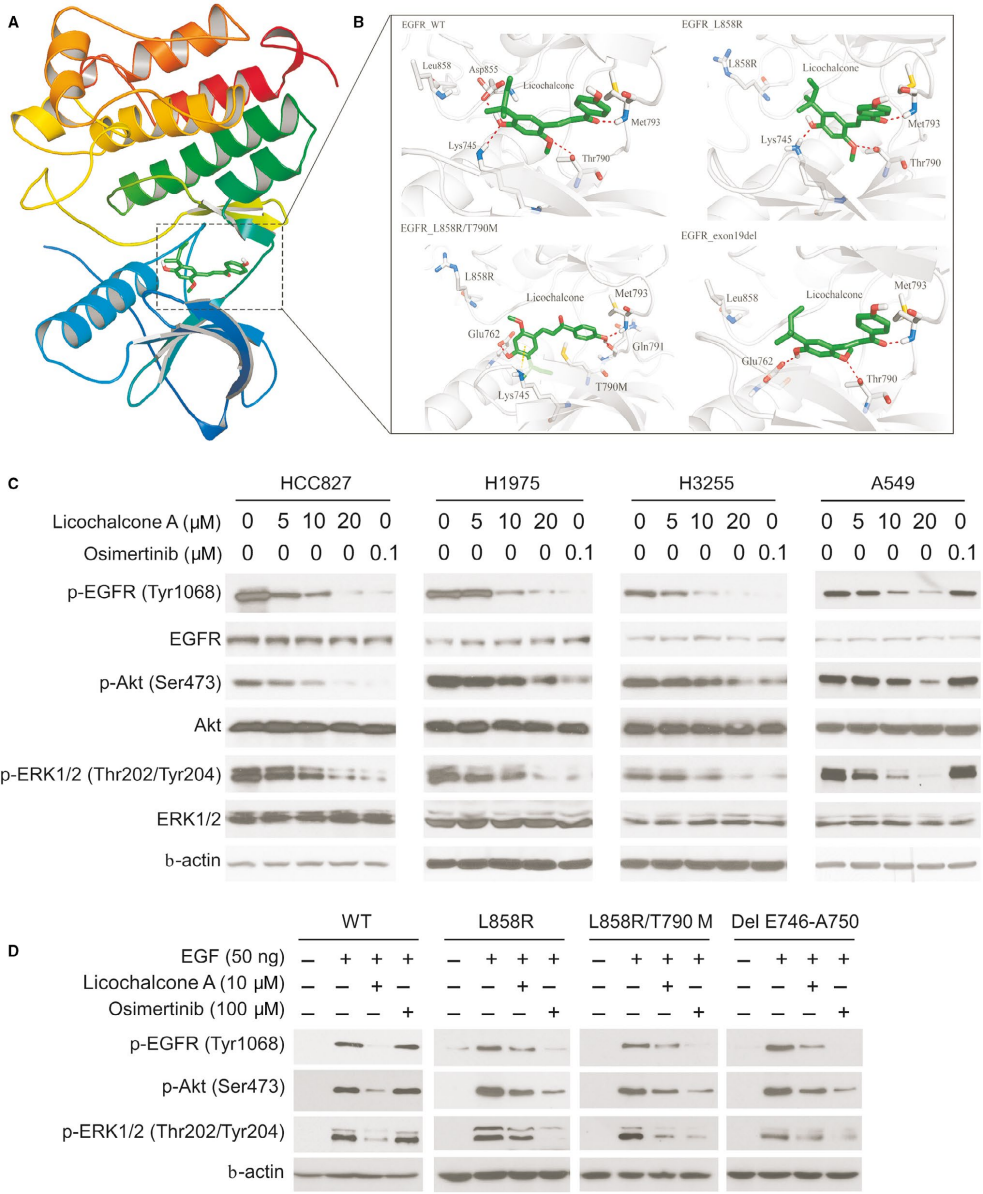
Licochalcone A suppresses EGFR signalling. A, B, The binding modes of licochalcone A with WT and mutant EGFRs. Cartoon representation of licochalcone A binding pocket in EGFR (A). Different binding modes of licochalcone A with 4 types of EGFR (B). The ligands were shown in the sticks, whereas proteins were depicted in cartoon representation with key residues indicated as grey sticks. Hydrogen bonds are shown as red dashed lines, and the cation‐π interaction is shown as yellow dashed line. Besides, the relating residues or ligands were well labelled. C, Licochalcone A inhibits EGFR signalling in NSCLC cells. The NSCLC cells were treated with licochalcone A and osimertinib, and whole‐cell extracts (WCE) were subjected to IB analysis. D, Ba/F3 cells stable cells were pre‐treated with licochalcone A or osimertinib for 2 h and then treated with EGF for 15 min. WCE was collected and subjected to IB analysis

To better understand the anti‐tumour mechanism of licochalcone A, we examined whether licochalcone A could affect EGFR signalling pathway. The in vitro EGFR kinase assays showed that either licochalcone A or osimertinib significantly suppressed the activity of the activating mutant EGFRs, including EGFR Del E746‐A750, EGFR L858R/T790M and EGFR L858R (Figure 2A‐C). Licochalcone A substantially inhibited the activation of the L858R and Del E746‐A750 mutants at the concentration of 5 μmol/L, whereas licochalcone A suppressed kinase activity of the EGFR L858R/T790M double mutant only after the concentration reached at 10 μmol/L (Figure 2A‐C). Although osimertinib blocked the activation of all of these three activating mutants at the concentration of 100 nmol/L, it only reduced the activity of WT EGFR by less than 30% at this dosage. In contrast, licochalcone A exhibited a more substantial inhibitory effect than that of osimertinib and reduced the EGFR activity up to 70% when the concentration reached at 20 μmol/L (Figure 2D). By incubation with the whole‐cell lysates from HCC827 (Figure 2E), H1975 (Figure 2F), H3255 (Figure 2G) and A549 (Figure 2H), we found that both WT and mutant EGFRs interacted with licochalcone A–conjugated Sepharose 4B beads. We also confirmed the in vitro pull‐down assay using the purified EGFR WT and mutant proteins. The results showed that licochalcone A binds with the purified EGFR WT and mutant proteins as expected (Figure S2). Moreover, the in vitro ATP competition assay showed that the binding efficacy of licochalcone A with EGFRs was decreased in the presence of ATP (Figure 3I‐L), suggesting that binding with licochalcone A might impair the binding between ATP and EGFRs and eventually result in the suppression of EGFR activity.

Molecular modelling showed that licochalcone was docked into the ATP‐binding pocket of EGFRs, including exon 19 deletion mutation, L858R single‐site mutation, L858R/T790M double mutations and wild‐type. As shown in Figure 3A,B, the docking poses suggested that licochalcone could form several interactions with WT EGFR and specific forms of mutated EGFR. Of note, the hydrogen bond with the backbone nitrogen of Met793 in the hinge region was shown both in the WT EGFR and the mutated EGFRs. However, the T790M mutation lost a hydrogen bond with the ligand because a hydrogen bond donor was preferred according to the binding modes of the other 3 types of EGFR. On the other hand, in the L858R/T790M mutations, licochalcone could interact with Lys745 through cation‐π interaction to improve its binding except through hydrogen bonds. Other hydrogen bonds between WT EGFR and licochalcone were shown, which were formed with the side chains of Lys745 and Asp855. The L858R mutation caused marginal variation of the pocket, and the binding pose did not change so much. The exon 19 deletion might have changed the shape of the pocket, in which licochalcone was predicted to interact with Met793, Thr790 and Glu762 by hydrogen bonding (Figure 3B). Our data indicate that licochalcone might be a good hit, especially for designing novel EGFR inhibitors with selectivity to different mutation types.

Immunoblotting analysis suggested that EGFR activity was decreased in response to licochalcone A treatment in NSCLC cells. However, licochalcone A can not significantly suppress the activity of WT EGFR at the dose of 5 μmol/L, which is consistent with our in vitro kinase assay that a higher concentration of licochalcone A was required for blocking of WT EGFR activity (Figure 3C). Akt and ERK kinases are two primary downstream targets of EGFR kinase. Our data indicated that both licochalcone A and osimertinib dramatically inhibited the phosphorylation of Akt and ERK1/2 in HCC827, H1975 and H3255 cells in a dose‐dependent manner (Figure 3C). However, only licochalcone A, but not osimertinib, inhibited WT EGFR, Akt and ERK1/2 phosphorylation in A549 cells. We further examined EGFR signalling in Ba/F3 stable cells carrying various EGFRs, including Del E746‐A750, L858R or L858R/T790M mutants, and WT. The immunoblotting data showed that licochalcone A or osimertinib exhibited a similar inhibitory effects on Del E746‐A750, L858R and L858R/T790M mutant‐expressing stable cell lines (Figure 3D). Consistently, licochalcone A exhibited a stronger inhibitory effect on WT EGFR than that of osimertinib (Figure 3D). Taken together, our data indicate that licochalcone inhibits the activation of both wild‐type and mutant EGFRs.

### 3.3 Licochalcone A induces apoptosis in NSCLC Cells

**Figure 4:**
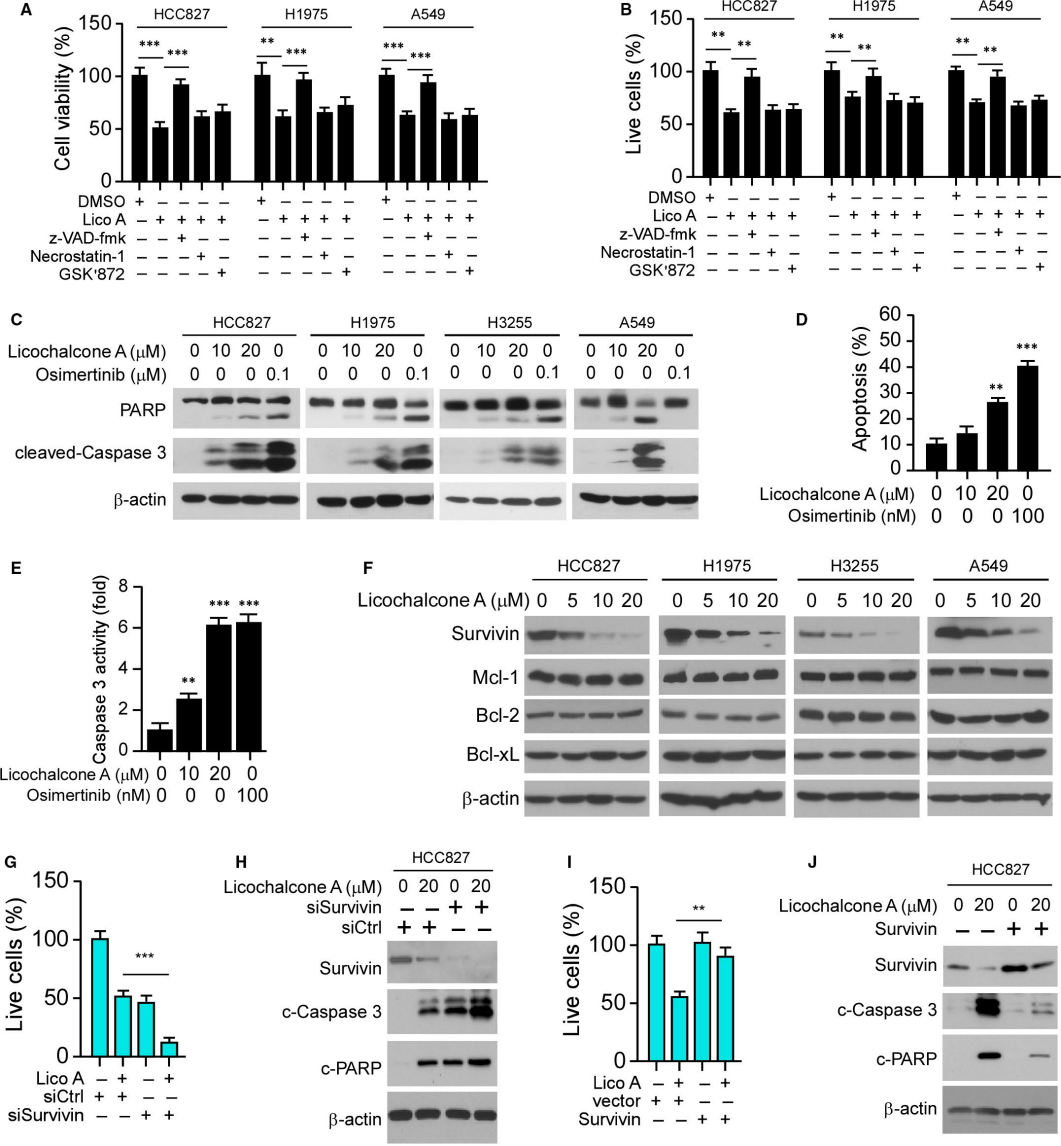
Licochalcone A induces apoptosis in NSCLC cells. A and B, NSCLC cells were pre‐treated with z‐VAD‐fmk, GSK’873 or necrostatin‐1 for 4 h, then maintained in licochalcone A containing medium for 48 h, and cell viability and live cell population were examined by MTS (A) and trypan blue exclusion assay (B). **P < .01, ***P < .001. C, HCC827, H1975, H3255 and A549 cells were treated with licochalcone A or osimertinib for 48 h, and WCE was subjected to IB analysis as indicated. D, Flow cytometry analysis of apoptotic HCC827 cells with licochalcone A or osimertinib treatment for 48 h. **P < .01, ***P < .001. E, Normalized caspase 3 activity in licochalcone A‐ or osimertinib‐treated HCC827 cells for 48 h. **P < .01, ***P < .001. F, IB analysis of NSCLC cells with licochalcone A treatment for 48 h. G, HCC827 cells transfected with siSurvivin and/or treated with licochalcone A, and then subjected to trypan blue exclusion assay for live cell population analysis. ***P < .001. H, Cells treated in G were subjected to IB analysis. I, HCC827 cells transfected with Survivin and/or treated with licochalcone A and subjected to trypan blue exclusion assay for live cell population analysis. **P < .01. J, The cells treated in I were subjected to IB analysis

HCC827, H1975 and A549 cells were pre‐treated with inhibitors of apoptosis and necroptosis, such as z‐VAD‐fmk, GSK’873 or necrostatin‐1. The MTS data showed that only z‐VAD‐fmk rescued licochalcone A–induced cell death (Figure 4A), which is confirmed by the trypan blue exclusion assay (Figure 4B). These results indicate that licochalcone A promoted apoptosis in NSCLC cells. Treatment with licochalcone A or osimertinib promoted the protein level of cleaved caspase 3 and cleaved PARP in HCC827, H3255 and H1975 cells (Figure 4C). However, the osimertinib‐induced apoptosis in WT EGFR expression A549 cell was compromised when compared with that of licochalcone A–treated A549 cells (Figure 4C). Also, licochalcone A–induced apoptosis was further validated by caspase 3 activity and flow cytometry analysis (Figure 4D,E). Treatment with licochalcone A decreased the protein level of survivin, but not Bcl‐2, Bcl‐xL or Mcl‐1, robustly in either WT or mutant EGFR‐expressing NSCLC cells (Figure 4F). To examine whether survivin plays a key role in licochalcone A–induced apoptosis, we overexpressed survivin in HCC827 (Figure 4G‐J) and H1975 (Figure S3A‐D) cells. The result showed that overexpression of survivin compromised licochalcone A–induced apoptosis, whereas knockdown of survivin enhanced apoptosis (Figure 4H,J, Figure S3B,D). Consistently, overexpression of survivin increased the live cell population even in the presence of licochalcone A (Figure 4G,I, Figure S3A,C). These results suggest that licochalcone A induces cell death by promoting apoptosis, and survivin plays a key role in this process.

### 3.4 Survivin is translationally regulated by licochalcone A in NSCLC cells

**Figure 5:**
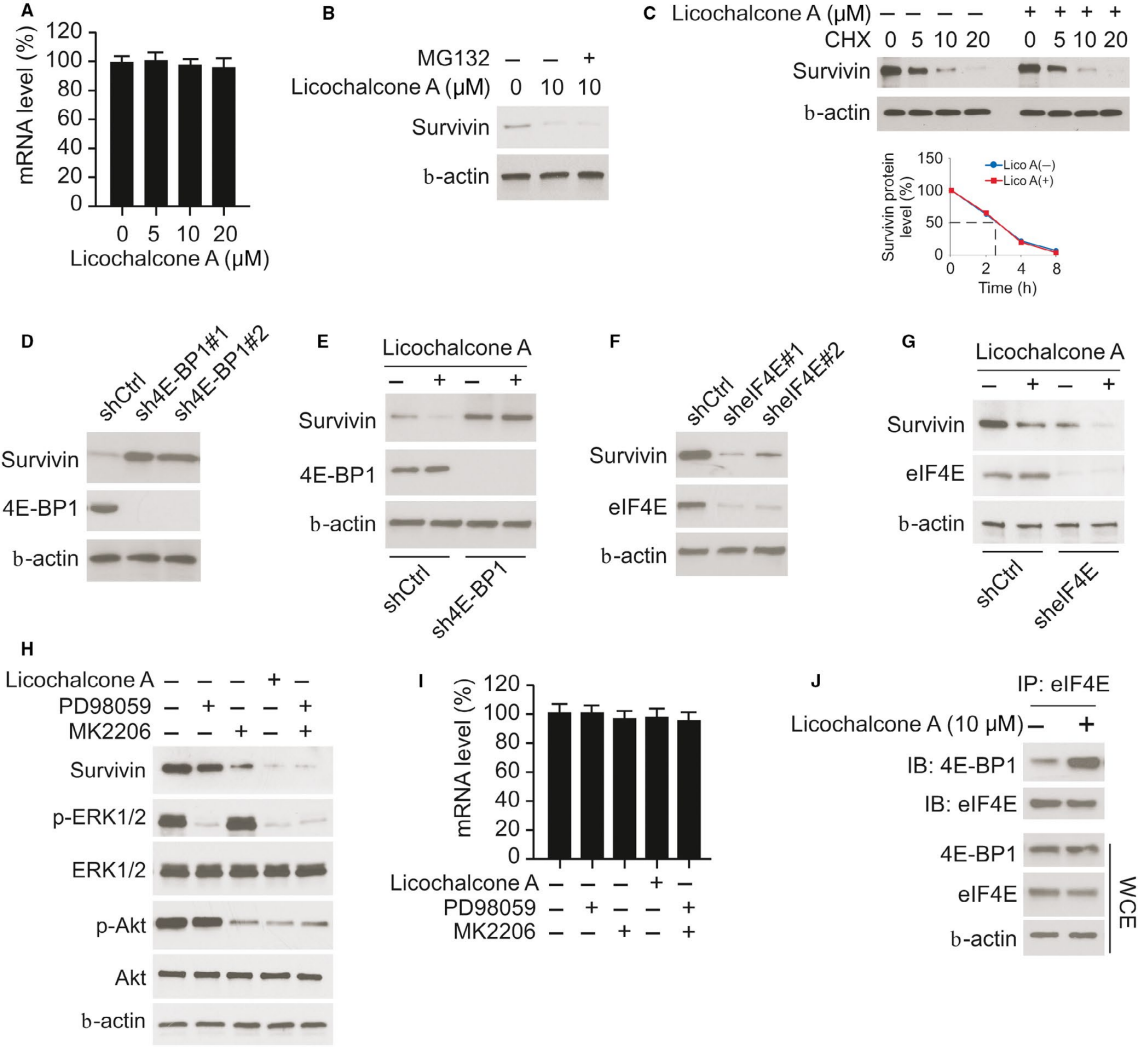
Licochalcone A translationally regulates survivin in NSCLC cells. A, HCC827 cells were treated with licochalcone A, and the mRNA level of survivin was analysed by quantitative RT‐PCR. B, IB analysis of HCC827 cells treated with licochalcone A and MG132. C, HCC827 cells were treated with licochalcone A for 24 h and co‐cultured with cycloheximide (CHX) for various time‐points, and WCE was subjected to IB analysis (top). The line chart shows the half‐life of survivin from IB analysis (bottom). D, IB analysis of survivin in HCC827‐shGFP and HCC827‐sh4E‐BP1 stable cells. E, IB analysis of HCC827‐shGFP and HCC827‐sh4E‐BP1 stable cells treated with licochalcone A or DMSO. F, IB analysis of survivin in HCC827‐shGFP and HCC827‐sheIF4E stable cells. G, HCC827‐shGFP and HCC827‐sheIF4E stable cells were treated with DMSO or licochalcone A and subjected to IB analysis. H and I, HCC827 cells were treated with ERK1/2 inhibitor PD98059, Akt inhibitor MK2206, licochalcone A, or a combination of PD98059 and MK2206 for 24 h, and WCE was subjected to IB analysis (H) or quantitative RT‐PCR analysis (I). J, Co‐immunoprecipitation (co‐IP) and IB analysis of HCC827 cells treated with DMSO or licochalcone A

To further confirm that EGFR signalling is required for survivin expression in NSCLC cells, we examined the survivin protein level with EGFR inhibitor treatment. We found that osimertinib inhibited the phosphorylation of EGFR in HC827, H1975 and H3255 cells robustly. Consistently, the protein level of survivin was decreased in these EGFR mutant cells, but not in A549 with wild‐type EGFR (Figure S4A). Furthermore, knockdown of EGFR with siRNA reduced survivin expression robustly in HCC827 and H1975 cells (Figure S4B). We further generated EGFR knockout stable cells in A549 cells using sgRNA. The data revealed that overexpression of EGFR activation mutant Del E746‐A750 or L858R/T790M restored survivin protein level in A549 cells with EGFR knockout (Figure S4C). The qRT‐PCR data revealed that licochalcone A did not affect the mRNA level of survivin (Figure 5A). Moreover, incubation with MG132 failed to rescue licochalcone A–induced down‐regulation of survivin (Figure 5B). Also, cycloheximide chase assay showed that the protein degradation rate was similar in licochalcone A‐ and DMSO‐treated HCC827 cells (Figure 5C). These data indicate that the reduction of survivin expression was not caused by the transcription suppression or protein degradation. Strikingly, we found that knockdown of 4E‐BP1 by shRNA, which resulted in the activation of cap‐dependent translation increased the protein level of survivin (Figure 5D). Furthermore, depletion of 4E‐BP1 compromised licochalcone A–induced down‐regulation of survivin protein (Figure 5E). Conversely, suppression of cap‐dependent translation by stable expression of eIF4E shRNA decreased the expression of survivin (Figure 5F), and treatment with licochalcone A in eIF4E‐deficient stable cells caused a much more potent reduction of survivin protein when compared with that of eIF4E proficient shCtrl stable cells (Figure 5G). These data suggest that impairment of cap‐dependent translation is involved in licochalcone A–induced decrease in survivin protein. To determine which signalling pathway is associated with this process, we pharmacologically inhibited the major downstream kinases of EGFR, Akt and ERK1/2, by small molecular compound MK2206 and PD98059, respectively. The data showed that licochalcone A and the combination of MK2206 and PD98059 decreased survivin much more robust than that in MK2206 or PD98059 treated alone (Figure 5H), indicating that licochalcone A–induced survivin reduction was largely mediated by attenuation of Akt and ERK1/2 activity. Consistently, the mRNA level of survivin was similar in all of these treated groups (Figure 5I). Additionally, treatment with licochalcone A reduced the phosphorylation of 4E‐BP1 in NSCLC cells (Figure S4D). Importantly, the co‐immunoprecipitation (co‐IP) data demonstrated that licochalcone A enhanced the interaction between eIF4E and 4E‐BP1 (Figure 5J), which further confirmed the inhibition of cap‐dependent translation in licochalcone A–treated NSCLC cell. Together, our data suggest that down‐regulation of Akt and ERK signalling by licochalcone A decreased survivin expression through inhibition of cap‐dependent translation in NSCLC cells.

### 3.5 Licochalcone A suppresses xenograft tumour growth in vivo

**Figure 6:**
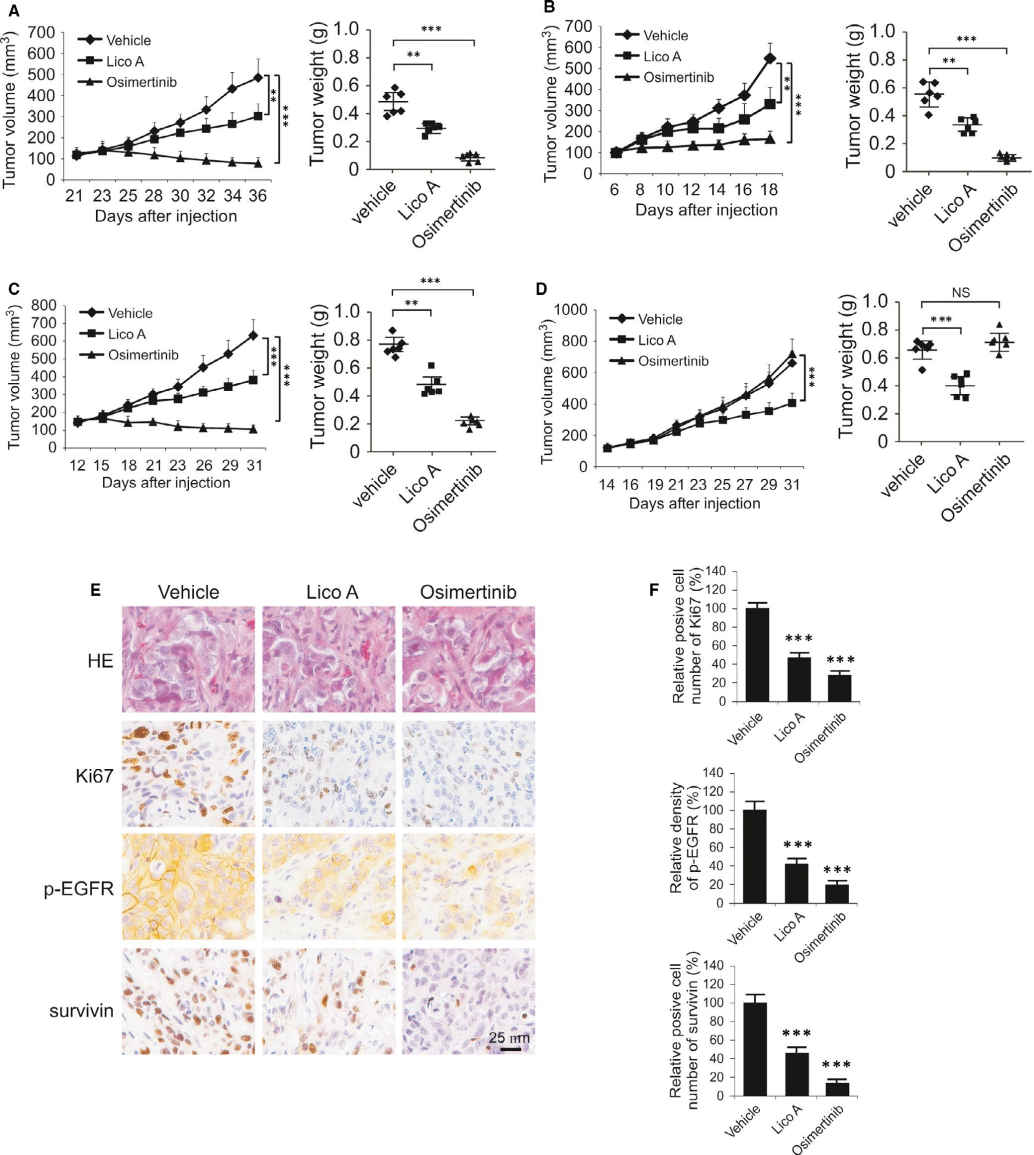
Licochalcone A inhibits xenograft tumour growth. A‐d, HCC827 (A), H1975 (B), H3255 (C) and A549 (D) xenograft tumours were treated with vehicle control, licochalcone A, or osimertinib, tumour volume (left) and tumour weight (right) were recorded. **P < .01, ***P < .001. ns, not statistically significant. E, IHC staining analysis of p‐EGFR, Ki67 and survivin in HCC827 xenograft tumours. F, Quantitative analysis of p‐EGFR, Ki67 and survivin from E. ***P < .001

We next examined the in vivo anti‐tumour activity of licochalcone A using the xenograft mouse model. When tumour volume reached around 100 mm^3^, treatment with licochalcone A, osimertinib or vehicle control was initiated. Our data indicated that every 2 days dosing of licochalcone A delayed the tumour growth of HCC827 (Figure 6A), H3255 (Figure 6B) and H1975 (Figure 6C) xenografts. Osimertinib blocked tumour growth in the HCC827 and H3255 xenograft tumours, but failed in A549 (Figure 6D) xenograft with EGFR WT. In contrast, licochalcone A reduced tumour size significantly. Moreover, the phosphorylated EGFR, ki67 and total protein level of survivin were examined by immunohistochemical analysis. As shown in Figure 6E,F, licochalcone A suppressed EGFR kinase activity. Consistently, the protein levels of Ki67 and survivin were reduced with licochalcone A or osimertinib treatment. These results suggest that licochalcone A inhibits the in vivo tumour growth of both osimertinib‐sensitive and osimertinib‐resistant xenografts.

## 4 DISCUSSION

With the development of diagnosis and treatment, especially the successful clinical application of EGFR TKI, the overall survival of NSCLC patients has improved significantly during the past years.^24^, ^25^, ^26^ Currently, osimertinib is the only irreversible third‐generation EGFR‐TKI approved for the treatment of EGFR‐activating mutations and the EGFR T790M mutation in patients with EGFR oncogene addiction.^27^, ^28^ Despite the documented anti‐tumour activity of osimertinib, most patients develop resistance within 2 years. Reports indicate that the emergence of new somatic mutation of EGFR, HER2/MET amplification, RAS/MAPK or PI3K/Akt signalling activation, histological/phenotypic transformation to small‐cell lung cancer and novel fusion events are associated with osimertinib acquired resistance.^3^, ^5^, ^29^, ^30^, ^31^, ^32^ However, over 30%‐40% of acquired resistance mechanisms are still elusive. Thus, there is an urgent demand to develop novel anti‐tumour drugs or identify new therapeutic targets that can complement current EGFR‐targeted therapy. In the present study, we demonstrated that licochalcone A exhibited significant anti‐tumour efficacy against NSCLC cells. Licochalcone A binds with EGFR and inhibits EGFR activity in vitro, ex vivo and in vivo. Thus, licochalcone A acts as an EGFR inhibitor and is expected to have beneficial effects in the treatment of NSCLC.

Survivin plays a critical role for cancer cell survival and metastasis in multiple human cancer cells.^33^, ^34^ Previous studies have shown that survivin is highly expressed in human cancers, including lung,^35^ ovarian,^36^ cervical^37^ and colorectal cancer,^38^ glioblastoma^39^ and T cell lymphoma.^40^ A recent study demonstrated that survivin is overexpressed on cancer stem cells and required for maintaining cancer stem cell properties.^41^ Furthermore, survivin could be processed and presented by dendritic cells and activates the CTL response in vitro or in a murine melanoma model in vivo.^42^, ^43^ Survivin protein expression is involved in the progression of NSCLC^35^ and decreases survivin by anti‐tumour compound T21‐inhibited NSCLC cell growth and T21‐induced apoptosis.^44^, ^45^ Moreover, metformin promotes survivin degradation through AMPK/PKA/GSK‐3β‐axis, which reduces the cell viability of NSCLC cells.^46^ This evidence indicates survivin is a fantastic target in cancer treatment. Indeed suppression of transactivation of survivin through direct binding to its promoter, the small molecule inhibitors YM‐155 and terameprocol (EM‐1421) decreased survivin protein and induced apoptosis in human cancer cells.^47^, ^48^, ^49^ Additionally, targeting survivin enhanced tumour chemo‐ and radio‐sensitivity.^50^, ^51^, ^52^ Our data demonstrated that licochalcone A translationally regulates survivin expression, but exhibits no significant effect on mRNA level and protein stability. Importantly, licochalcone A–mediated survivin down‐regulation is partly dependent on the suppression of EGFR downstream Akt and ERK1/2 signallings, which is consistent with the previous report that reduces survivin protein by brexpiprazole overcomes EGFR TKI resistance in lung and pancreatic cancer.^53^

Overall, this study investigated the anti‐tumour efficacy of licochalcone A in NSCLC cells. Through suppression of EGFR signalling and decrease in survivin expression, licochalcone A exhibited profound antitumour potential in either EGFR WT or mutant NSCLC cells. Our studies provided new insights into the role of licochalcone A in cancer treatment and suggested licochalcone A might be a therapeutic agent against this devastating disease.

## CONFLICT OF INTEREST

The authors declare that they have no conflict of interest.

## AUTHOR CONTRIBUTIONS

**Feng Gao:** Conceptualization (equal); data curation (equal); formal analysis (equal); funding acquisition (equal); methodology (equal). **Ming Li:** Conceptualization (equal); data curation (equal); formal analysis (equal); investigation (equal); methodology (equal). **Xinfang Yu:** Conceptualization (equal); data curation (equal); formal analysis (equal); investigation (equal); methodology (equal). **Wenbin Liu:** Data curation (equal); formal analysis (equal); investigation (equal). **Li Zhou:** Data curation (equal); formal analysis (equal); investigation (equal). **Wei Li:** Conceptualization (equal); data curation (equal); formal analysis (equal); funding acquisition (equal); investigation (equal); methodology (equal); project administration (equal).

## Supporting information

Fig S1

Fig S2

Fig S3

Fig S4

Supplementary Material

## Data Availability

The data sets generated and analysed during this study are available from the corresponding author on reasonable request.
