# Supplementary figures and images for: Licochalcone A inhibits EGFR signalling and translationally suppresses survivin expression in human cancer cells
Source: J Cell Mol Med. 2020 Nov 27;25(2):813–26. doi: 10.1111/jcmm.16135 (PMC7812290; doi:10.1111/jcmm.16135)

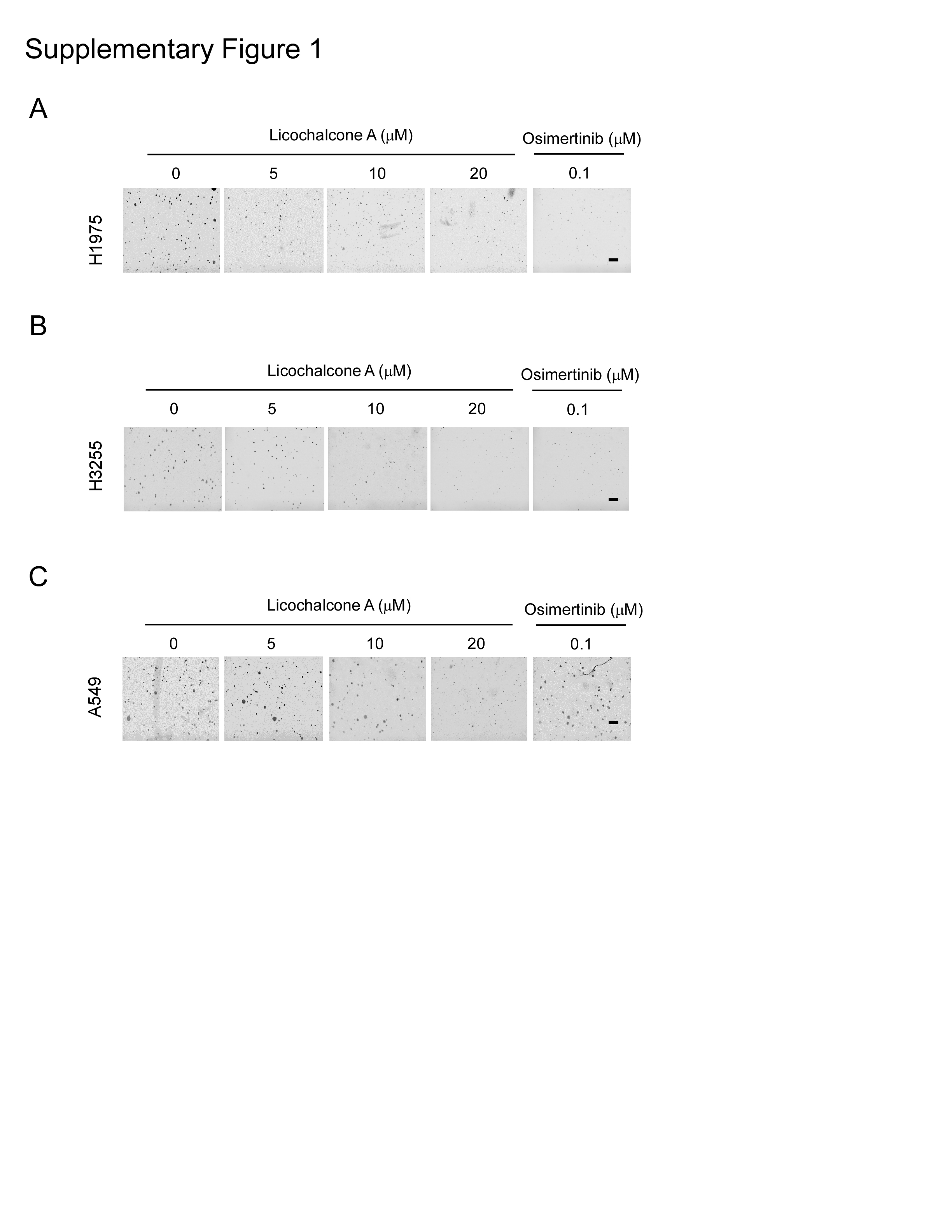

Supplement: Supplementary file 1 — Fig S1 [file JCMM-25-813-s001.jpg]

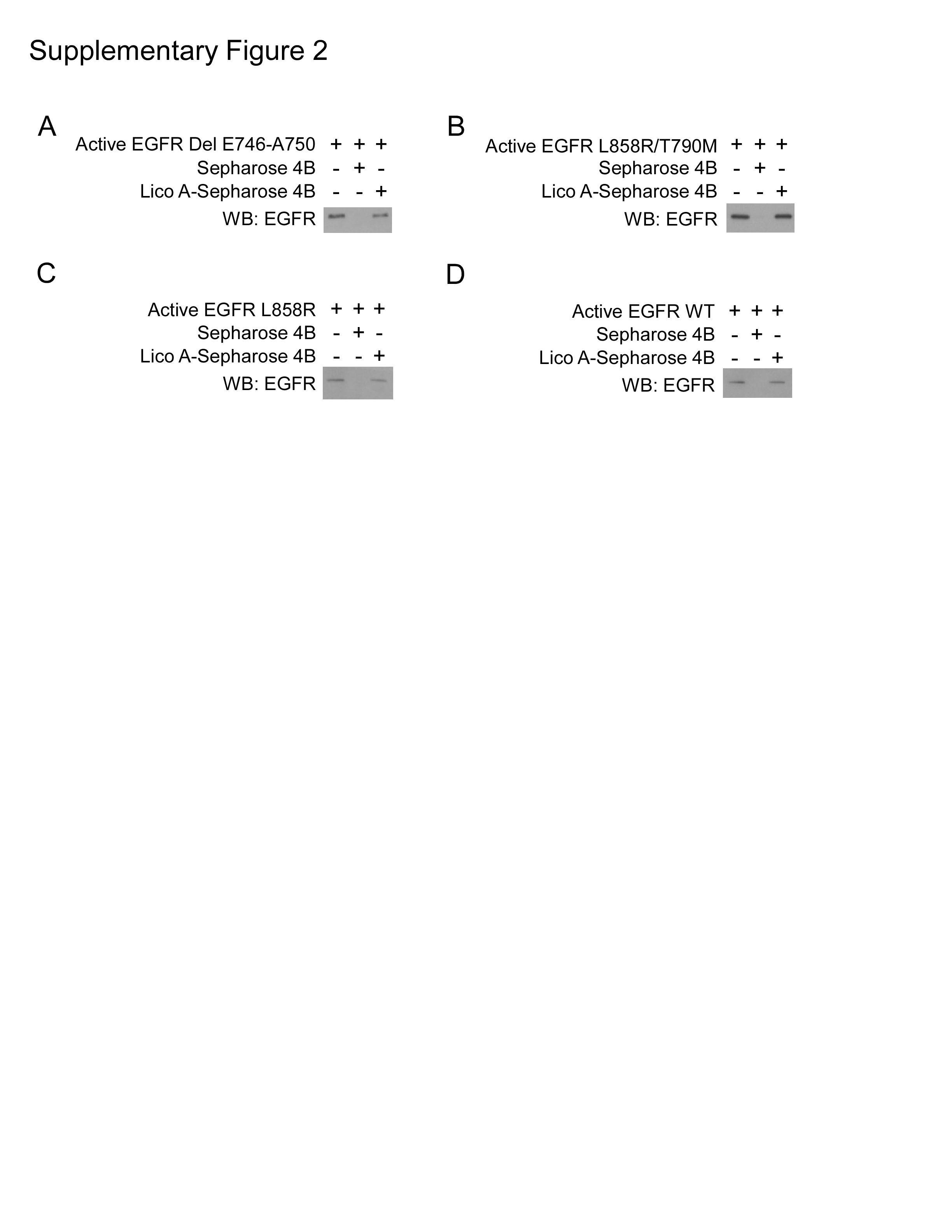

Supplement: Supplementary file 2 — Fig S2 [file JCMM-25-813-s002.jpg]

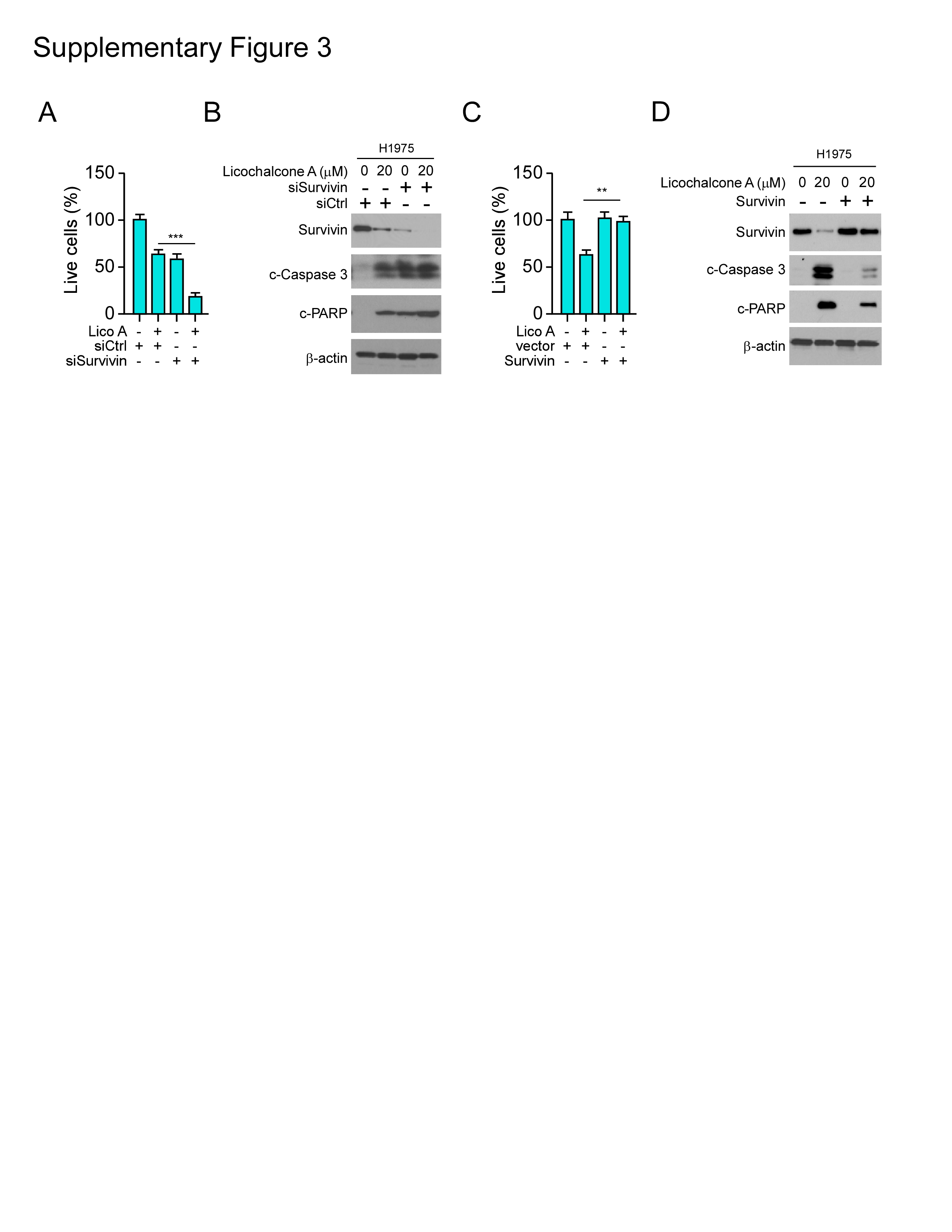

Supplement: Supplementary file 3 — Fig S3 [file JCMM-25-813-s003.jpg]

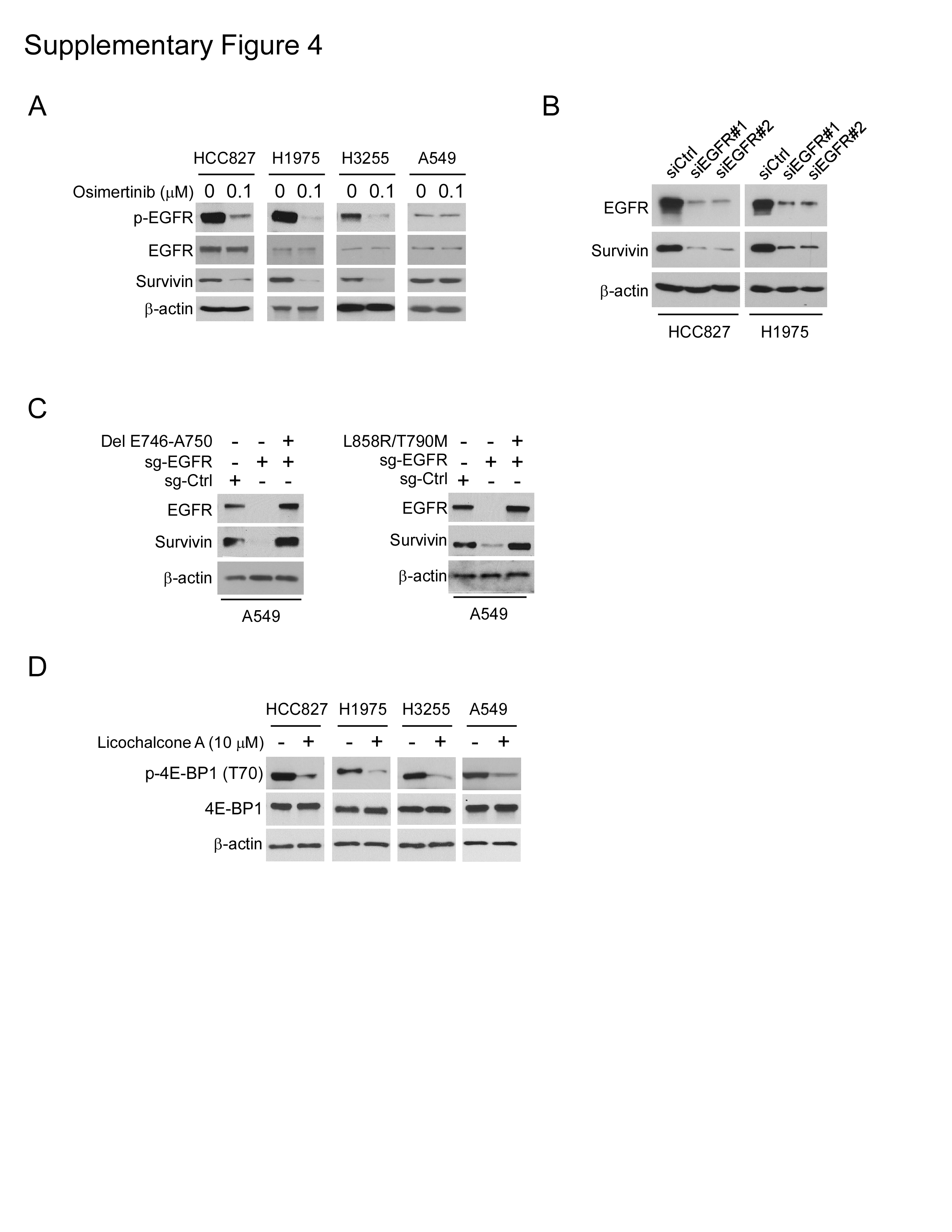

Supplement: Supplementary file 4 — Fig S4 [file JCMM-25-813-s004.jpg]
