# Supplementary material for: Licochalcone A inhibits EGFR signalling and translationally suppresses survivin expression in human cancer cells
Source: J Cell Mol Med. 2020 Nov 27;25(2):813–26. doi: 10.1111/jcmm.16135 (PMC7812290; doi:10.1111/jcmm.16135)
Supplement: Supplementary file 5 — Supplementary Material [file JCMM-25-813-s005.docx]

**Supplementary figure legends**

Supplementary Figure 1. Licochalcone A inhibits the anchorage-independent cell growth of non-small cell lung cancer (NSCLC) cells. The H1975 (A), H3255 (B), and A549 (C) cells were treated with licochalcone A or osmertinib and subjected to soft agar assay. Scale bar, 500 μm.

Supplementary Figure 2. Licochalcone A binds with EGFR WT and mutants. Purified active EGFR kinases, including EGFR Del E746-A750 (A), EGFR L858R/T790M (B), EGFR L858R (C), and EGFR WT (D) were incubated with licochalcone A-Sepharose 4B beads (Sepharose 4B beads only as control) overnight at 4°C. The beads were washed and boiled with loading buffer and subjected to immunoblotting (IB) analysis.

Supplementary Figure 3. Survivin plays a crucial role in Licochalcone A-induced apoptosis. A, Trypan blue exclusion assay analysis of live cell population of H1975 cells transfected with siSurvivin and/or treated with licochalcone A. ****p*<0.001. B, The cells treated in A were subjected to IB analysis with the indicated antibodies. C, Trypan blue exclusion assay analysis of live cell population of H1975 cells transfected with Survivin and/or treated with licochalcone A. ***p*<0.01. D, The cells treated in C were subjected to IB analysis with the indicated antibodies.

Supplementary Figure 4. EGFR signaling is required for survivin expression in NSCLC cells. A, NSCLC cells were treated with osimertinib for 24 h, whole cell lysates were subjected to immunoblotting (IB) analysis. B, HCC827 and H1975 cells were transfected with siCtrl or siEGFR, whole cell lysates were subjected to immunoblotting (IB) analysis. C, A549 cells were transfected with sgRNA targeting EGFR and selected with 1 ug/ml puromycin for 10 days. The sgEGFR cells were transfected with EGFR E746-A750 or L858R/T790M, whole cell lysates were subjected to IB analysis. D, NSCLC were treated with licochalcone A for 24 h, whole cell lysates were subjected to IB analysis.
